# Simultaneous engineering of the surface (oxygen/amorphous carbon) and interface (amorphous carbon/ZnO) of ZnO using a one-spoon amorphous carbon deposition technique

**DOI:** 10.1016/j.jare.2026.01.057

**Published:** 2026-01-30

**Authors:** Jimyeong Park, Minseo Kim, Changhyun Jin, Kyu Hyoung Lee, Myung Sik Choi

**Affiliations:** a Department of Nano & Advanced Materials Science and Engineering, Kyungpook National University, Sangju 37224, the Republic of Korea; b Department of Materials Science and Engineering, Yonsei University, Seoul 03722, the Republic of Korea

**Keywords:** ZnO, Amorphous carbon, Quenching, Heat treatment, Gas sensors

## Abstract

*Introduction:* Surface and interface engineering of metal oxide semiconductors is important for improving their gas-sensing performance.

*Objectives:* This study aimed to simultaneously engineer the surface and interface of ZnO porous nanosheets (PNSs) using a simple one-spoon amorphous carbon deposition (OSaCD) technique and to investigate its effect on NO_2_ gas-sensing performance.

*Methods:* Amorphous carbon/ZnO porous nanosheets (aC/ZnO PNSs) were synthesized using an innovative OSaCD coating technique combined with water quenching during flame chemical vapor deposition (FCVD). The coating characteristics were tuned by adjusting simple processing parameters.

*Results:* The incorporation of amorphous carbon (aC) enhanced NO_2_ gas-sensing performance at both the surface and interfacial levels. At the surface, this improvement arose from interactions among oxygen species, the target gas, and aC, whereas at the interface, it was associated with the aC/ZnO junction. An enhanced sensor response of 18.8 ± 1.2 was achieved at an NO_2_ concentration of 4 ppm at 200 °C. Notably, the sensor was capable of detecting NO_2_ at concentrations as low as 200 ppb. This enhanced performance was attributed to the expansion of the surface electron depletion layer and the reduction of oxygen vacancies at the interface.

*Conclusion:* Owing to the intrinsic fluidity of the OSaCD process, this strategy is broadly applicable and can potentially be extended beyond specific material systems, morphologies, compositions, or degrees of crystallinity.

## Highlights

• This study introduces a novel one-spoon amorphous carbon deposition (OSaCD) method.
• Amorphous carbon/ZnO porous nanosheets were synthesized using the water-quenching technique.
• This method utilizes the synergistic effect of the surface and interface of amorphous carbon.

## Graphical abstract

Conceptual diagram illustrating the principle of increased resistance at the surface and interface in aC/ZnO PNSs.

**Figure:**
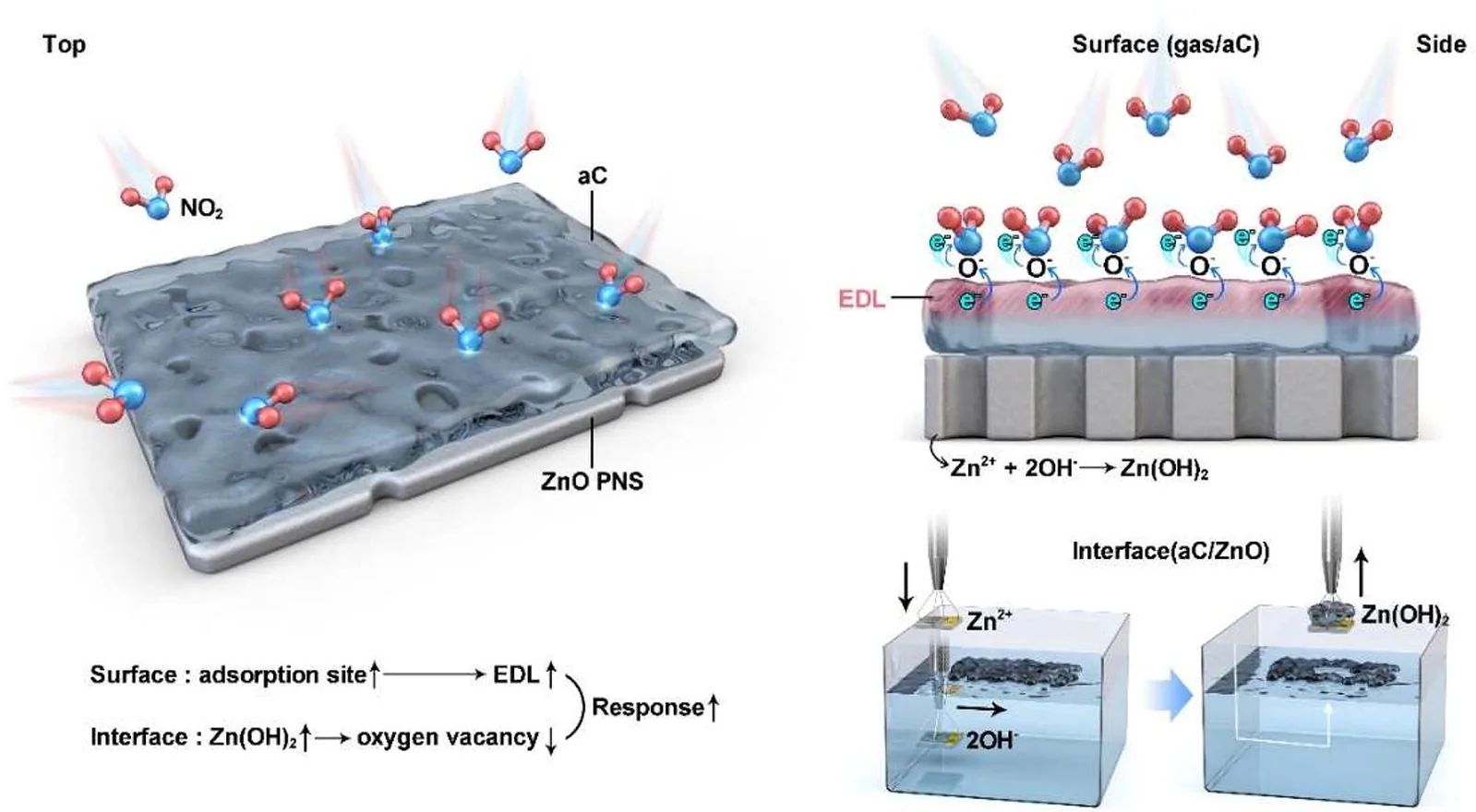

## Introduction

One of the most distinctive features of semiconductor gas sensors is that most reactions occur at the semiconductor surface, which is in direct contact with the target gas [1], [2], [3]. Consequently, surface properties play a critical role in gas-sensing behavior. For example, Xiong et al. [4] reported that various point defects can form on the surface of SnO_2_. These include oxygen-based defects such as oxygen vacancies, double-ionized and single-ionized oxygen vacancies, bridging and sub-bridging oxygen vacancies, oxygen antisites, and Sn^4+^–oxygen vacancy associates. Tin-based defects, including tin interstitials, vacancies, and antisites, were also observed. Among these defects, oxygen vacancies overwhelmingly influence gas sensing, contributing more than 82% of the total effect, corresponding to 70 out of 85 defect types. Despite their importance, controlling surface defects generally requires additional pre- and post-treatment steps. These include doping, chemical reduction, irradiation, and heat treatment. Such processes increase cost and complexity and require additional time, space, equipment, and vacuum systems.

For instance, Li et al. [5] controlled oxygen vacancies in single-crystal In_2_O_3_ at room temperature (RT) using different oxygen concentrations. A low oxygen concentration of 0.02% O_2_ in Ar increased conductance, whereas a higher concentration of 0.04% O_2_ in Ar decreased conductance. However, this process required vacuum conditions to maintain smooth carrier gas flow and UV illumination pretreatment to clean the surface. Similarly, Gu et al. [6] synthesized long, single-crystalline Au-functionalized ZnO nanowires using a wet-chemical reduction method. These nanowires exhibited high gas-sensing responses and short response and recovery times toward organic vapors, including 2-propanol, acetone, ethanol, methanol, and benzene. However, the synthesis required multiple precursors, such as NaOH, KOH, Zn(NO_3_)_2_·6H_2_O, HAuCl_4_·2H_2_O, and C_6_H_5_O_7_Na_3_·2H_2_O. The use of these reagents increased both processing time and cost. Kim et al. [7] reported ZnO nanofibers with superior gas-sensing properties toward H_2_S gas at 350 °C, achieving a response of 150 to 10 ppm H_2_S. This performance was attributed to increased oxygen vacancies and Zn interstitials generated by electron beam irradiation. However, high-energy irradiation at 1 MeV and multiple dose treatments of 50, 100, and 150 kGy resulted in substantial economic and spatial costs. In addition, Nemufulwi et al. [8] synthesized fibrous ZnFe_2_O_4_ using a combustion method and achieved a response of 192 to 90 ppm acetone at 120 °C. This process required extensive annealing steps with heating rates of 5, 10, and 15 °C/min. As a result, the method was both time-consuming and space-intensive.

To address these limitations, this study employs the quenching principle of flame chemical vapor deposition (FCVD) [9], [10], [11]. This process eliminates the need for pre- and post-treatment steps and does not require vacuum systems. It also offers advantages in cost efficiency, stability, and portability. Using this method, amorphous carbon (aC) was coated onto ZnO porous nanosheets (PNS) under various heat treatment conditions, as shown in Table 1 [12], [13], [14], [15], [16], [17], [18], [19], [20], [21], [22], [23], [24], [25], [26], [27], [28], [29], [30], [31]. The optimized gas-sensing performance toward NO_2_, which is particularly suitable for ZnO, was achieved through tailored heat treatments, as summarized in Table 2 [32], [33], [34], [35], [36], [37], [38].

**Table 1:** Comparison of conditions required for coating.

Material source	Method	Time required (s)		Equipment	Environmental and temperature	Cost (Equip-$)	Vacuum (Torr)	Ref
		Carbonization time	Coating times					
Acetone	CVD	5,400		Quartz tube reactor		∼1,000	100 (working pressure)	12
Acetylene	CVD	30–3,600		Furnace, centrifuge	Argon, 725 °C	∼1,000	10^−3^–10^−6^	13
Acetylene	CVD	3,600		Autoclave,tube furnace	500 °C	∼1,000	10^−3^–10^−6^	14
Acetylene gas	PECVD	120–600		PECVD chamber	Plasma	∼4,000	7.5 × 10^-7^	15
Carbon target	ECR plasma sputtering		7,200	ECR sputter	Argon plasma	∼100,000	7.5 × 10^−6^	16
CH_4_	CVD	10,800		Furnace	1250 °C	∼1,000	10^−3^–10^−6^	17
CNT	Solvothermal	600	172,800	Sonicator Autoclave	180 °C, 600 °C	∼1000	760	18
Ethylene	RF-PECVD	10,800		Sputter	RT ∼ 600 °C	∼100,000	4 × 10^−3^ (working pressure)	19
Ferrocene		172,800		Oven	220 °C	∼700	760	20
Glucose	Hydrothermal		10,800	Autoclave, centrifuge	Argon, 180 °C	∼2500	10^−3^–10^−6^	21
Glucose	Solvothermal	7,200	43,200	Sonicator, Autoclave	190 °C, 450 °C	∼1000	760	22
Glucose	Hydrothermal	7,200	14,400	Sonicator, Autoclave	Argon, 160 °C, 800 °C	∼1200	10^−3^–10^−6^	23
Graphite target	Sputtering		30–12,600	Magnetron sputter, vacuum chamber	Argon plasma	∼30,000	3.75 × 10^−5^	24
Graphite target	Sputtering		1,200–3,600	Magnetron sputter	Argon plasma	∼30,000	3 × 10^−5^	25
Graphite target	Sputtering		18,000	Magnetron sputter	Argon plasma	∼30,000	3 × 10^−5^	26
Graphite target	Sputtering		14,400	Magnetron sputter	Argon plasma	∼35,000	5 × 10^−5^	27
Pluronic F127	−	14,400	86,400	Oven	600 °C	∼700	10^−3^	28
Propylene	CVD	36,000		CVD reactor	700 °C	∼1,000	10^−3^–10^−6^	29
PVP		14,400	43.200	14,400	Nitrogen, 750 °C	∼800	10^−3^–10^−6^	30
Pyrrole monomer vapor		7,200	300	Oven	Agone, 400 °C	∼500	10^−3^–10^−6^	31
Flame	Water quenching	∼15	∼5	FCVD	Air, RT	40	760	Our work

**Table 2:** Examples of ZnO showing the best selectivity for NO_2_ gas.

Material	Response (NO_2_)/Concentration	Temperature	Highest response (other gas)/Concentration	Temperature	Ref
ZnO	11./3 ppm	RT (UV)	< 2.5/100 ppm	RT (UV)	32
ZG-600	13.86/100 ppm	100 °C	< 3/1000 ppm	150 °C	33
rGO-ZnO	6.77/10 ppm	RT	< 3/100 ppm	RT	34
rGO/ZnO	3.21/100 ppm	RT	< 1.5/100 ppm	RT	35
CM@ZnO	7.18/3 ppm	RT (25 °C)	< 2/10 ppm	RT (25 °C)	36
CNT-ZnO	1.023/10 ppm	300 °C	1.016/10 ppm	300 °C	37
Z-SWCNTs	7.14/5 ppm	RT	< 3/5 ppm	RT	38
CM@ZnO	7.18/3 ppm	RT (25 °C)	< 2/10 ppm	RT (25 °C)	39
aC/ZnO	17.2/4 ppm 9.65/2 ppm 8.15/1 ppm 6.00/600 ppb 5.31/200 ppb	200 °C	−	−	Our work

This study introduces a novel one-spoon amorphous carbon deposition (OSaCD) technique. In this approach, aC is floated on water and rapidly deposited onto ZnO substrates using the quenching principle. This method enhances the depth of the electron depletion layer (EDL) by increasing gas adsorption sites at the oxygen–aC or target gas–aC interface. At the same time, it reduces oxygen vacancies at the aC/ZnO interface. The OSaCD approach represents a competitive surface treatment technology capable of coating thin films onto semiconductor oxide nanostructures, regardless of morphology, composition, or crystallinity. This method provides a promising pathway for future gas-sensing technologies.

## Experimental section

### Synthesis of ZnO PNSs

A precursor solution was prepared by dissolving 0.02 mol of Zn(NO_3_)_2_·6H_2_O in 180 mL of a 2:1 mixture of deionized (DI) water and ethanol. The solution was stirred for 30 min. Subsequently, 0.03 mol of urea (RegentPlus, 99.5%, Sigma-Aldrich) was added, and stirring was continued for an additional 30 min. The resulting mixture was transferred into a 100 mL Teflon-lined autoclave. It was heated at a rate of 10 °C/min to 180 °C and maintained at this temperature for 24 h. After the reaction, the autoclave was allowed to cool to RT. The resulting white precipitate was collected by centrifugation and washed three times with ethanol and DI water. The product was then collected using a pressure-reducing filter and dried at 60 °C for 8 h. The dried powder was subsequently heat treated at 550 °C for 4 h. Finally, the ZnO porous nanosheets (PNSs) were drop coated onto a patterned alumina substrate. The substrate was heated on a hot plate at 100 °C.

### Coating of aC

An alumina crucible with dimensions of 50 cm in width, 75 cm in depth, and 11 cm in height was filled with 20 mL of water. An aC film was formed on the water surface using a 15 s FCVD process. During deposition, the flame served as the carbon precursor. The flame length was controlled at 45 ± 5 mm and exhibited a characteristic reddish emission. Each deposition cycle produced an aC domain with an approximate area of 4 × 4 mm^2^. The incident angle between the flame and the water surface was maintained at 40 ± 3°. The temperature of the water reservoir was regulated at 10 ± 3 °C. The substrate temperature during deposition was maintained at 25 °C. The previously synthesized ZnO PNSs on the alumina substrate were carefully held using a grabber. The substrate was immersed in the water containing the aC layer and then lifted together with the aC film. After coating, the substrate was dried on a hot plate. The temperature was gradually increased to RT, 50 °C, 75 °C, and 100 °C. These steps corresponded to the original state, surface chemical modification, structural and interface optimization, and graphitization, respectively.

### Characterizations

Morphological characterization was carried out using scanning electron microscopy (SEM, Regulus 8220, Hitachi Ltd.) and transmission electron microscopy (TEM, Titan G2 ChemiSTEM Cs Probe, FEI Company). Energy-dispersive X-ray spectroscopy (EDS) attached to the TEM was employed to analyze the chemical composition. High-angle annular dark-field scanning transmission electron microscopy (HAADF-STEM)was used to examine the microstructure. The crystal structure and phase composition were investigated by X-ray diffraction (XRD) using an Empyrean diffractometer (PANalytical B.V.) with Cu Kα_1_ radiation at a wavelength of 1.540598 Å. X-ray photoelectron spectroscopy (XPS) measurements were performed using a NEXSA system (Thermo Fisher Scientific Co.) to analyze chemical bonding states.

### Gas sensing tests

The gas sensor device was placed inside a custom-built gas sensing chamber. The back plate of the chamber was heated to control the operating temperature of the device. A 100 ppm mixed gas was injected into the chamber. For NO_2_ measurements, the test gas consisted of 100 ppm NO_2_ mixed with air. Dry gases, including H_2_S, C_2_H_5_OH, C_6_H_6_, C_7_H_8_, NH_3_, and H_2_, were prepared by mixing 100 ppm of each gas with N_2_. The gas concentration was adjusted to the desired level using a mass flow controller (MFC). The flow rate and gas concentration were monitored and controlled using an MR5000 system. The electrical resistance of the gas sensor was measured using a Keithley 3706A and a 2450 source meter. To generate a humid atmosphere, dry air mixed with gas from a bubble system was introduced at a specific concentration using an MFC. The humidity level was verified using a thermometer and relative humidity meter. The sensor response was defined as the ratio of resistance in air (R_a_) to resistance in the target gas (R_g_). For oxidizing gases, the response was calculated as R_g_/R_a_, whereas for reducing gases, it was calculated as R_a_/R_g_. The response and recovery times were determined based on the saturation point, corresponding to 90% of the total resistance change.

## Results and discussion

### Changes in the surface image of aC/ZnO PNS according to heat treatment.

Fig. 1 presents representative low- and high-magnification SEM images of the surface morphology of bare ZnO PNSs (Fig. 1a–b) and aC/ZnO PNS nanocomposites. The nanocomposites were synthesized by depositing aC followed by post-heat treatments at RT (aC/ZnO-RT; Fig. 1c–d), 50 °C (aC/ZnO-50; Fig. 1e–f), 75 °C (aC/ZnO-75; Fig. 1 g–h), and 100 °C (aC/ZnO-100; Fig. 1i–j). The bare ZnO PNSs exhibit a porous structure with pore sizes ranging from several tens of nanometers to less than 100 nm. These pores are distributed over a two-dimensional plate-like morphology, with some plates overlapping, as shown in Fig. 1a–b. Although the overall shape is roughly rectangular, many edges are irregularly connected. This feature increases the cross-sectional area and enhances gas adsorption. The porous and uneven surface of the ZnO PNSs, shown in Fig. 1a–b, provides a large number of adsorption sites. These sites arise from ledges and kinks, which are more effective for gas adsorption than terrace regions. After deposition of aC onto the ZnO PNSs, as shown in Fig. 1c–j, the formation of aC/ZnO PNS nanocomposites is clearly observed. No significant morphological differences are detected among the aC-coated samples subjected to different post-heat treatments. This uniformity results from the FCVD coating process, which employs a water-quenching method. During this process, the aC layer spreads two-dimensionally on the water surface before solidification, leading to homogeneous coverage. Consequently, the original pores of the bare ZnO PNSs are almost filled with the aC layer. This structure facilitates efficient transfer of mobile carriers between the aC and ZnO layers and leads to the formation of a double-layer configuration. As a result, aC exhibits two distinct interfaces. One interface is located at the surface, where aC interacts with oxygen. The second interface is formed at the contact between aC and ZnO. These dual interfaces play a crucial role in enhancing gas-sensing performance by promoting efficient interaction and carrier transport.

**Fig. 1:**
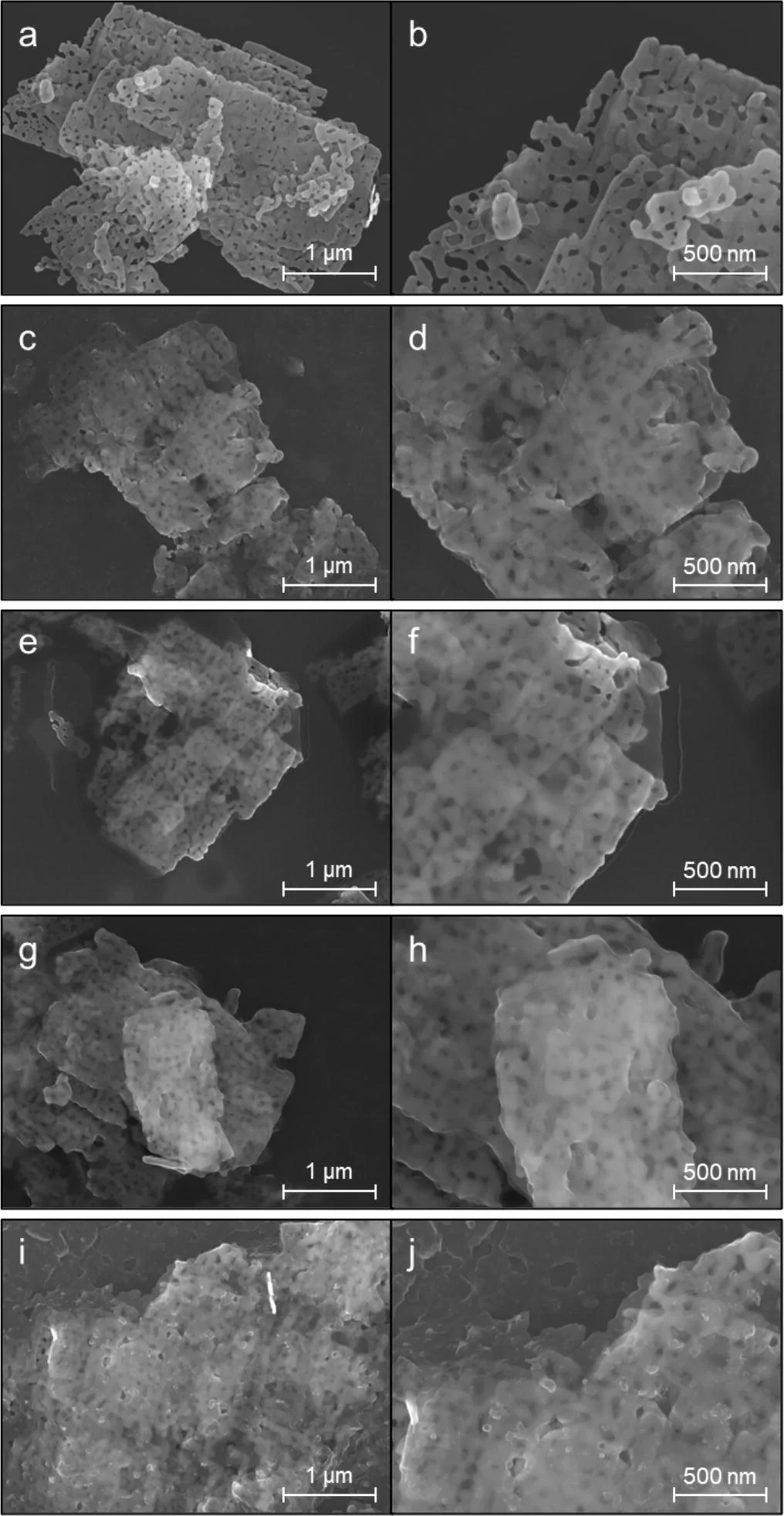
Low- and high-magnification SEM images of ZnO and aC/ZnO at different post-heat treatment temperatures: (a–b) bare ZnO, (c–d) aC/ZnO-RT, (e–f) aC/ZnO-50, (g–h) aC/ZnO-75, (i–j) aC/ZnO-100.

### Step-by-step characteristics of the OSaCD process

The deposition process produces aC with two critical interfaces. One interface is the surface layer of aC that is in contact with oxygen. The second interface is formed between aC and ZnO, which completes the heterostructure. This process is illustrated in Fig. 2, which presents the FCVD procedure. When the gaseous flame is rapidly cooled upon contact with water, it lacks sufficient time for regular crystal formation. As a result, the deposited aC layer exhibits an amorphous structure, as shown in Fig. 2a–b. During gas sensor fabrication, ZnO PNSs are first deposited onto an interdigitated electrode pattern. The aC layer is subsequently deposited by taking advantage of its fluid properties. As shown in Fig. 2c, the substrate is initially immersed in water in a region where no aC is present. It is then moved to an area containing the aC layer. The substrate is lifted from the water, thereby transferring the aC layer onto its surface, as illustrated in Fig. 2d. This deposition approach is referred to as the OSaCD method. The OSaCD technique represents a novel wet-coating approach for aC. It is not affected by the type of substrate or by the morphology, composition, or crystallinity of the ZnO PNSs. In addition, because the ZnO PNSs are already deposited on the substrate, the aC layer can be applied uniformly in a single step. This feature makes the process simple and efficient. The resulting aC/ZnO PNS nanocomposites were heat treated on a hot plate at temperatures ranging from RT to 100 °C to finalize the heterostructure. This method provides a practical advantage by enabling uniform coating of the entire substrate in one step, which allows for a wide range of applications. As summarized in Table 1, the FCVD method combined with water quenching offers significant advantages over conventional techniques, including reduced coating time, lower cost, and simplified process requirements.

**Fig. 2:**
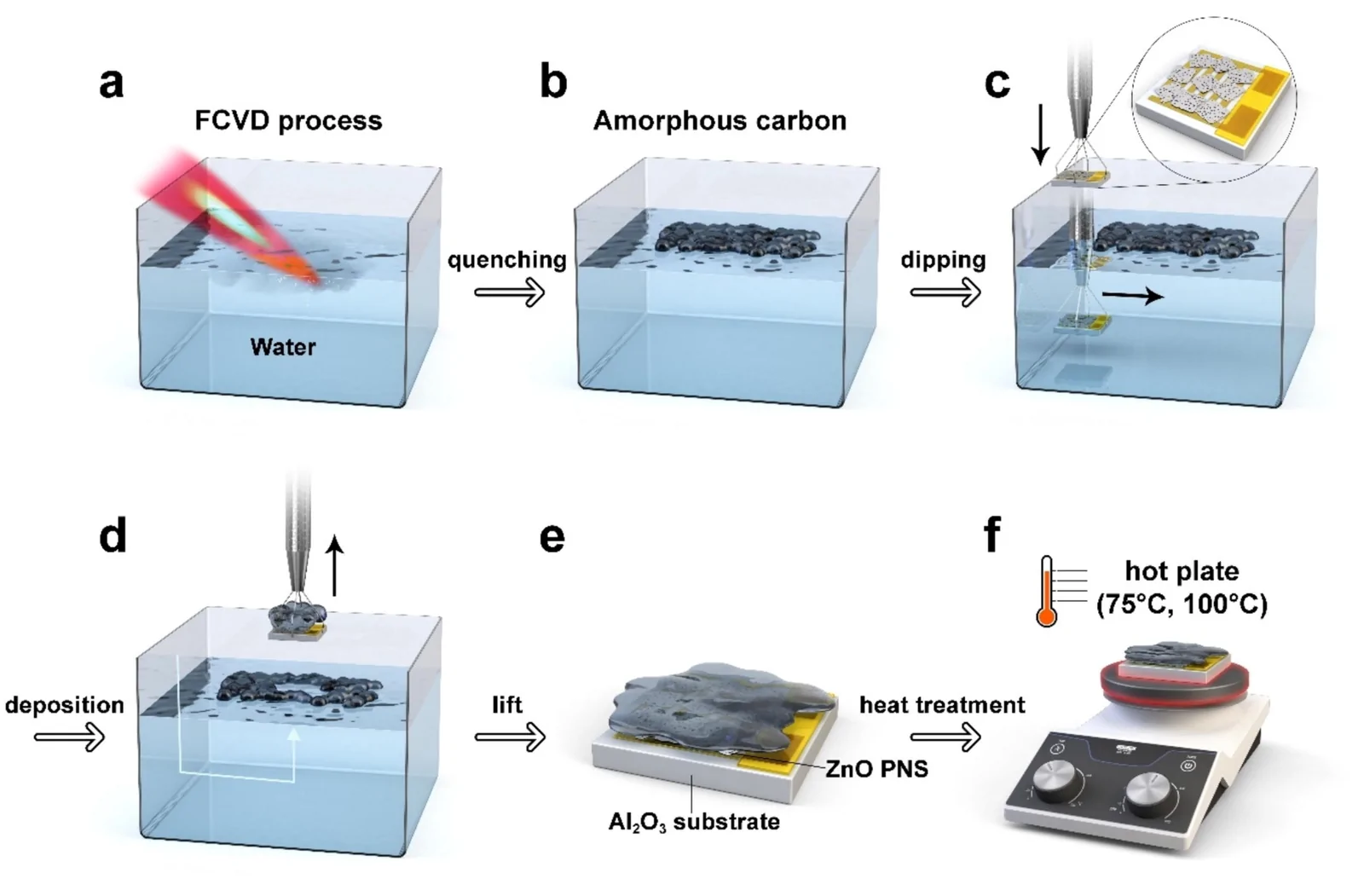
Schematic of the sequential synthesis of aC/ZnO PNSs using the OSaCD technique.

### Microstructure, crystal structure, and element distribution of aC/ZnO PNS

Fig. 3 presents TEM analysis used to confirm the proper layering of components in a heat-treated sample prepared using the method illustrated in Fig. 2. The low-magnification TEM image in Fig. 3a clearly shows that aC is uniformly coated on the two-dimensional ZnO PNSs. The aC layer forms a coating with an average thickness of approximately 44.92 ± 28.46 nm, as shown in the Supplementary Information (SI), Fig. S1. This uniform coating is achieved through the OSaCD method. Fig. 3b, 3c, and 3d reveal the composition of the resulting double-layer structure. The HAADF image in Fig. 3b and 3c indicate that the outermost layer corresponds to aC, while the inner layer is dominated by Zn and O. The line profile shown in Fig. 3d further confirms this elemental distribution. Depth profiling analysis supports these observations and demonstrates the expected elemental arrangement across the layers. Fig. 3e presents an HRTEM image that highlights the crystallinity of each region. The inner ZnO layer exhibits a single-crystal structure corresponding to the ZnO (002) plane. In contrast, the outer aC layer shows no long-range crystalline order. Fig. 3f displays a magnified HRTEM image of the ZnO (002) plane, while Fig. 3g shows the aC region. The corresponding FFT patterns of ZnO and aC are presented in Fig. 3h and 3i, respectively.

**Fig. 3:**
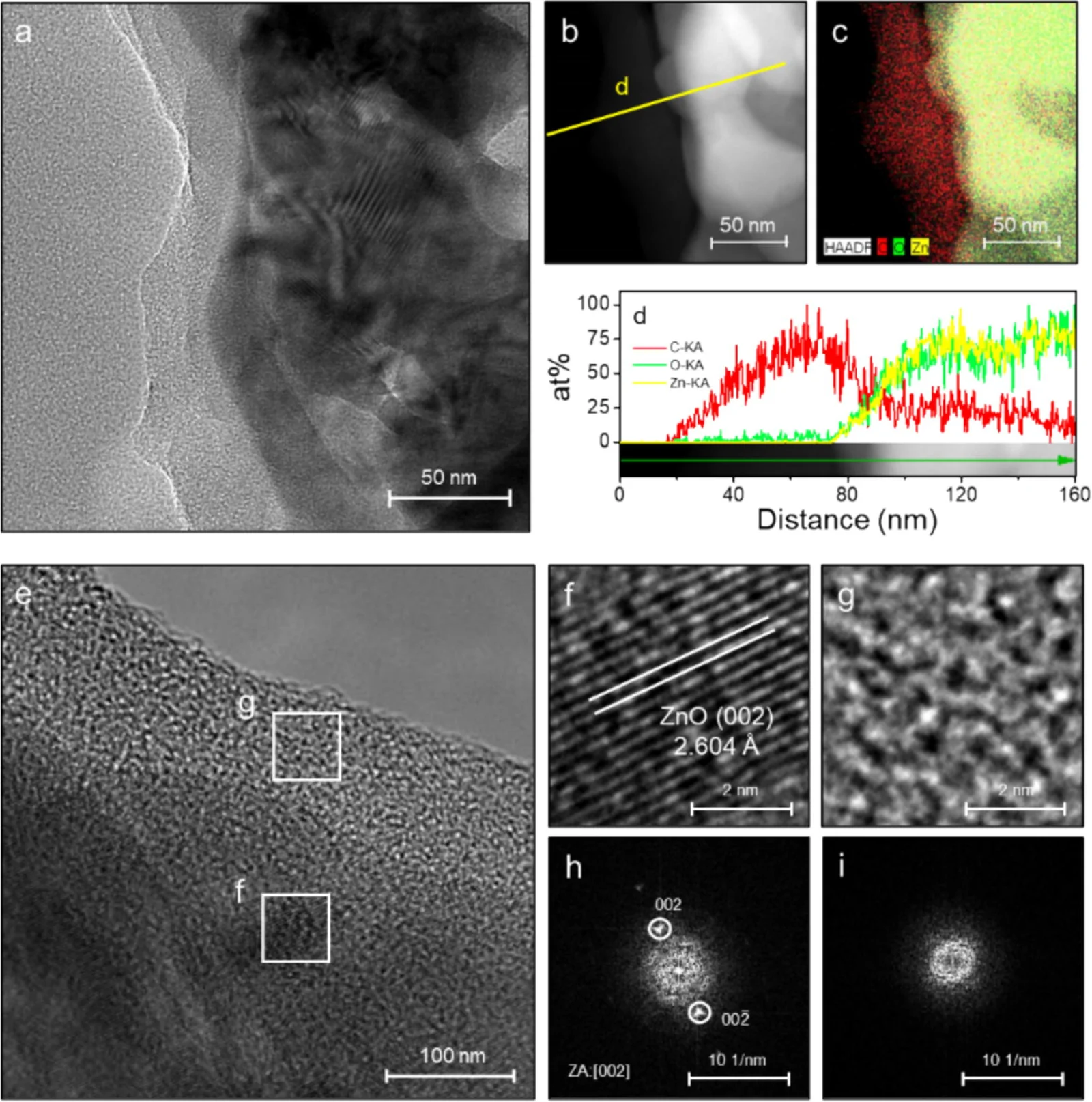
TEM and elemental mapping analysis of aC/ZnO-75: (a) low-magnification TEM image, (b) HAADF image, (c) elemental mapping, (d) line profile in HAADF, (e–g) HRTEM images of (f) ZnO and (g) aC regions, (h–i) corresponding FFT patterns of (h) ZnO and (i) aC.

### Analysis of various surfaces and interfaces of aC/ZnO PNS

Fig. 4a presents the XRD spectra used to examine the crystallinity of ZnO PNSs and aC/ZnO PNSs. For aC deposited on a silicon substrate, no crystalline features are observed, and only the substrate peak corresponding to the (111) plane is detected. In contrast, ZnO PNSs exhibit diffraction peaks at the (100), (002), and (101) planes. These peaks are consistent with JCPDS No. 36–1451 [39]. When the post-annealing temperature is increased to 75 and 100 °C, additional diffraction peaks corresponding to the (012), (104), and (110) planes appear in the aC/ZnO PNS nanocomposites, as shown in Fig. 4b. These peaks indicate changes in crystallinity that are not observed in the bare ZnO PNSs.

**Fig. 4:**
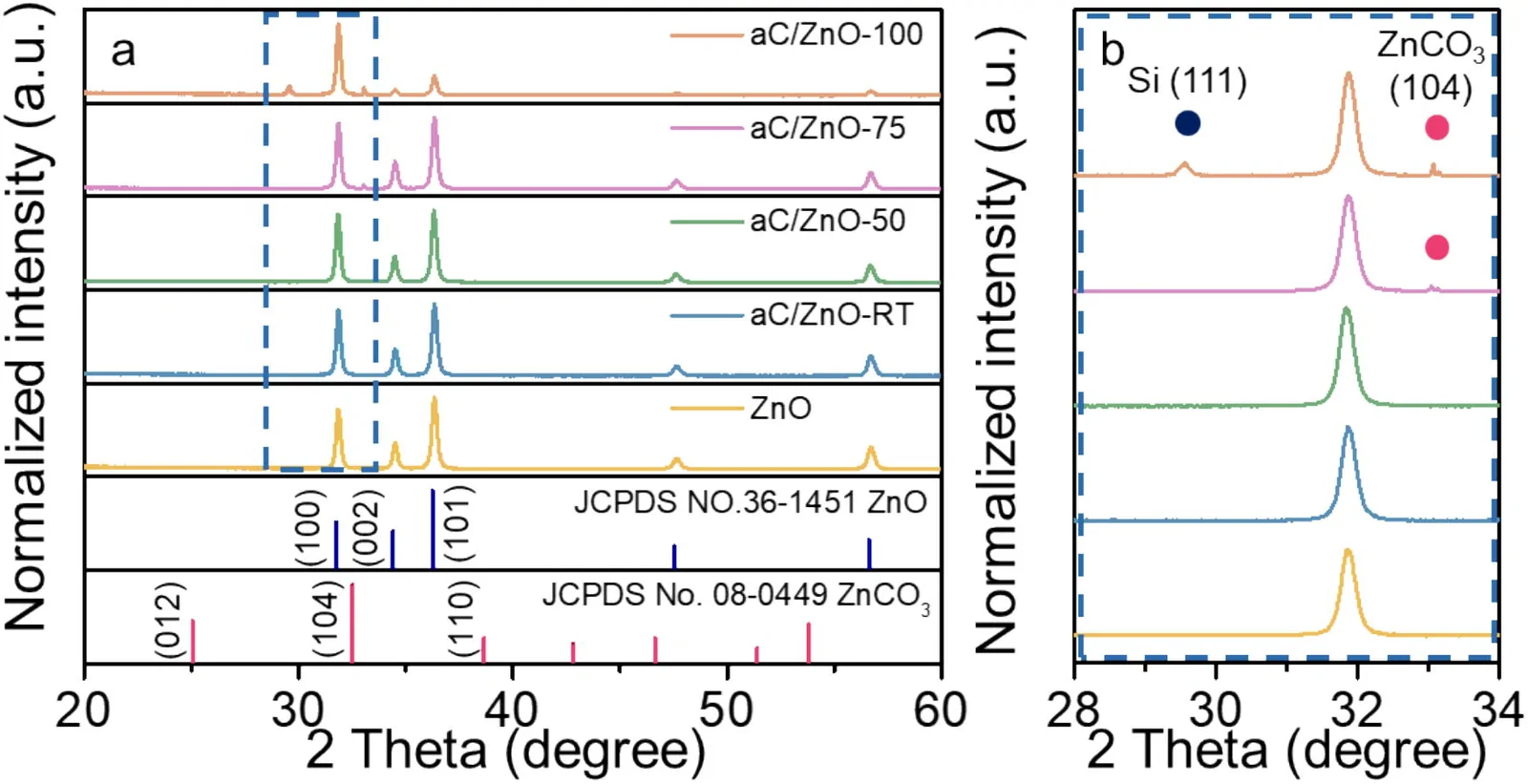
(a) XRD spectra of bare ZnO and aC/ZnO at different annealing temperatures, (b) XRD spectra at 2θ = 28°–34°.

Fig. 5 presents the XPS spectra used to evaluate the type and ratio of chemical bonding on the surfaces of ZnO PNSs, aC/ZnO-75, and aC samples. Bare ZnO exhibits a binding energy at 529.7 eV, whereas aC shows a binding energy at 532.8 eV. The aC/ZnO-75 sample displays a binding energy of 531.2 eV, as shown in Fig. 5a. This value lies between those of ZnO and aC, suggesting the presence of interactions at both the gas/aC surface and the aC/ZnO interface. The introduction of aC modifies the oxygen bonding states depending on the post-heat treatment conditions. This effect is observed in bare ZnO (Fig. 5b), aC/ZnO-RT (Fig. 5c), aC/ZnO-50 (Fig. 5d), aC/ZnO-75 (Fig. 5e), and aC/ZnO-100 (Fig. 5f). As shown in Fig. 5b–5f and Table S1, the oxygen lattice fraction reaches its highest values in aC/ZnO-50 at 74.2% and aC/ZnO-75 at 73.3%. In contrast, oxygen vacancies at 28.5% and chemisorbed oxygen at 12.1% are most pronounced in aC/ZnO-RT. These results indicate that post-heat treatment promotes the conversion of oxygen vacancies and chemisorbed oxygen into lattice oxygen [40], [41], [42]. Overall, Fig. 5 demonstrates that surface binding states are modified following aC deposition. This modification enables control over the electron distribution and mobility in ZnO, which is an n-type semiconductor.

**Fig. 5:**
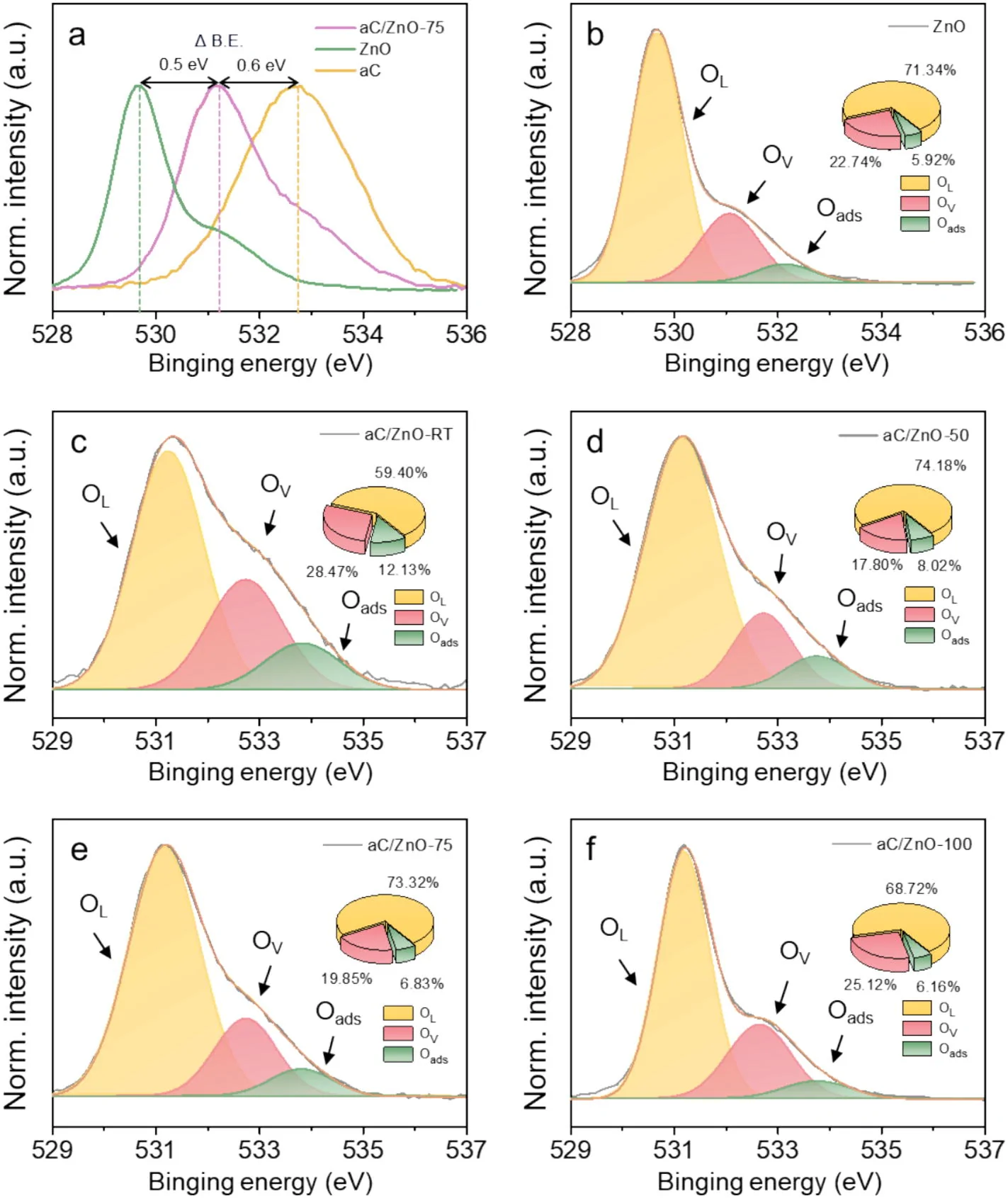
(a) XPS spectra of bare ZnO, aC/ZnO-75, and aC; (b–f) XPS spectra showing oxygen distribution in (b) bare ZnO and (c–f) aC/ZnO samples under different post-heat treatments.

Fig. 6 presents XPS analysis of aC bonding under different heat treatment conditions [43], [44], [45]. Four samples were examined: aC/ZnO (Fig. 6a), aC/ZnO-50 (Fig. 6b), aC/ZnO-75 (Fig. 6c), and aC/ZnO-100 (Fig. 6d). The results show that the four major bonding states, including sp^2^, sp^3^, C–O, and C=O, are distributed differently depending on the heat treatment temperature, as summarized in Fig. 6e. In general, sp^2^ and sp^3^ bonding dominates up to 75 °C. At 100 °C, C–O and C=O bonding becomes more prominent. Notably, 100 °C marks the onset of excessive changes, making it difficult to consistently attribute the observed trends to one or two specific causes. Nevertheless, the ratio of sp^2^ to sp^3^ bonding shows a continuous increase with increasing heat treatment temperature. The ratios increase sequentially from 0.66 to 0.72, 0.83, and 0.96, as shown in Fig. 6f.

**Fig. 6:**
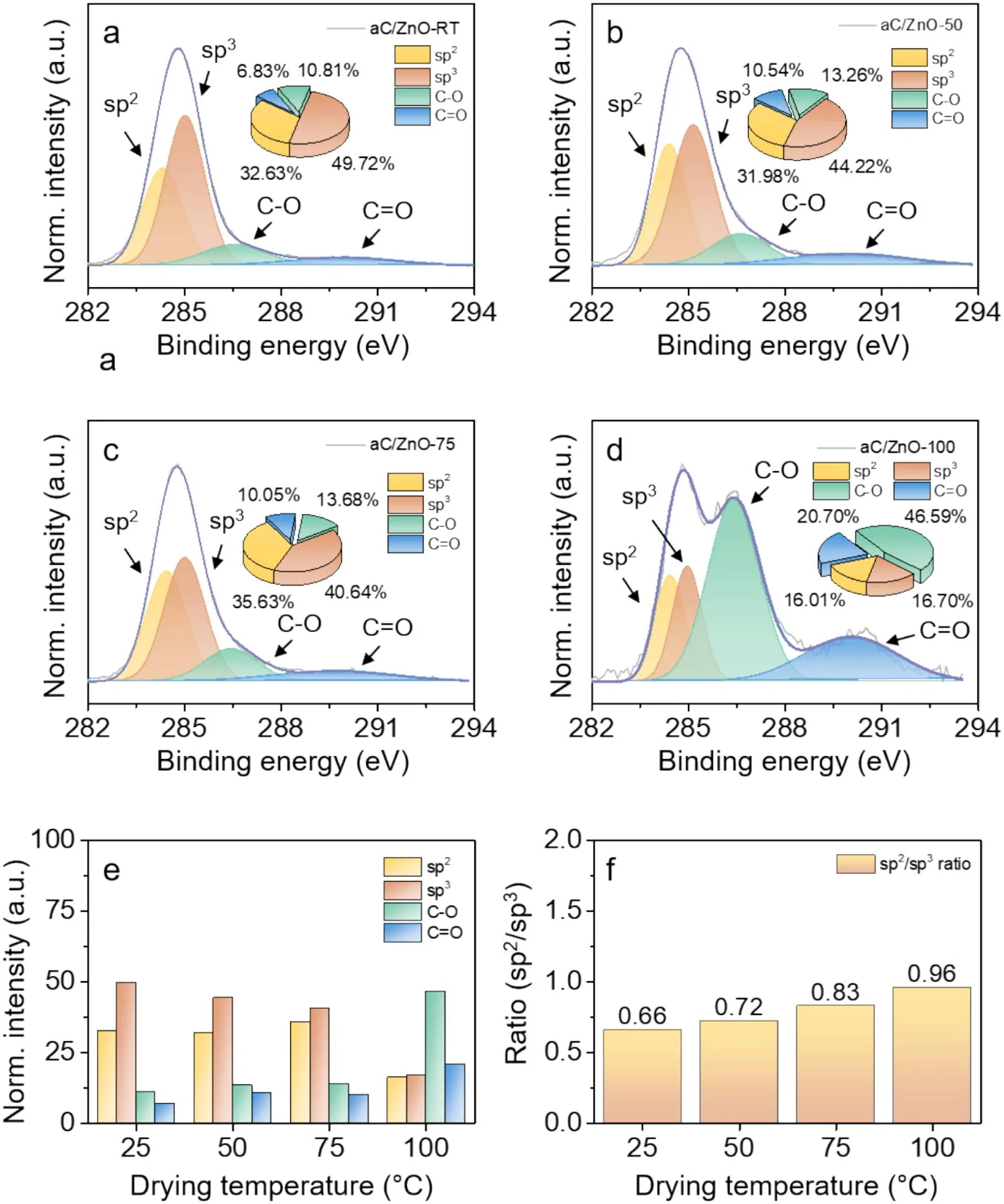
(a-d) XPS C1s spectra showing carbon distribution in (a) aC/ZnO-RT, (b) aC/ZnO-50, (c) aC/ZnO-75, and (d) aC/ZnO-100; (e) distribution of carbon bindings according to post-heat treatment; (f) sp^2^/sp^3^ ratio for each post-heat treatment.

Raman spectroscopy provides additional insight into the bonding states identified by XPS analysis. Fig. 7a and 7b clearly distinguish bare ZnO from aC/ZnO PNSs subjected to different post-heat treatment conditions. The aC/ZnO samples exhibit similar peak patterns, although the degree of disorder varies, as reflected by changes in the I_D_/I_G_ ratio of aC on the surface. According to previous reports [46], a higher I_D_/I_G_ ratio indicates a greater density of aC defects. Fig. 7c confirms that the aC/ZnO-75 sample exhibits the highest defect density. Although aC/ZnO-75 has the largest oxygen lattice fraction, as shown in Fig. 5 and SI Table S1, it also exhibits the highest defect density in the aC layer, particularly at the outer surface. This observation suggests that the outer aC layer is defect-rich. Such defects may enhance gas adsorption sites while simultaneously reducing oxygen vacancies and chemisorbed oxygen beneath the aC layer.

**Fig. 7:**
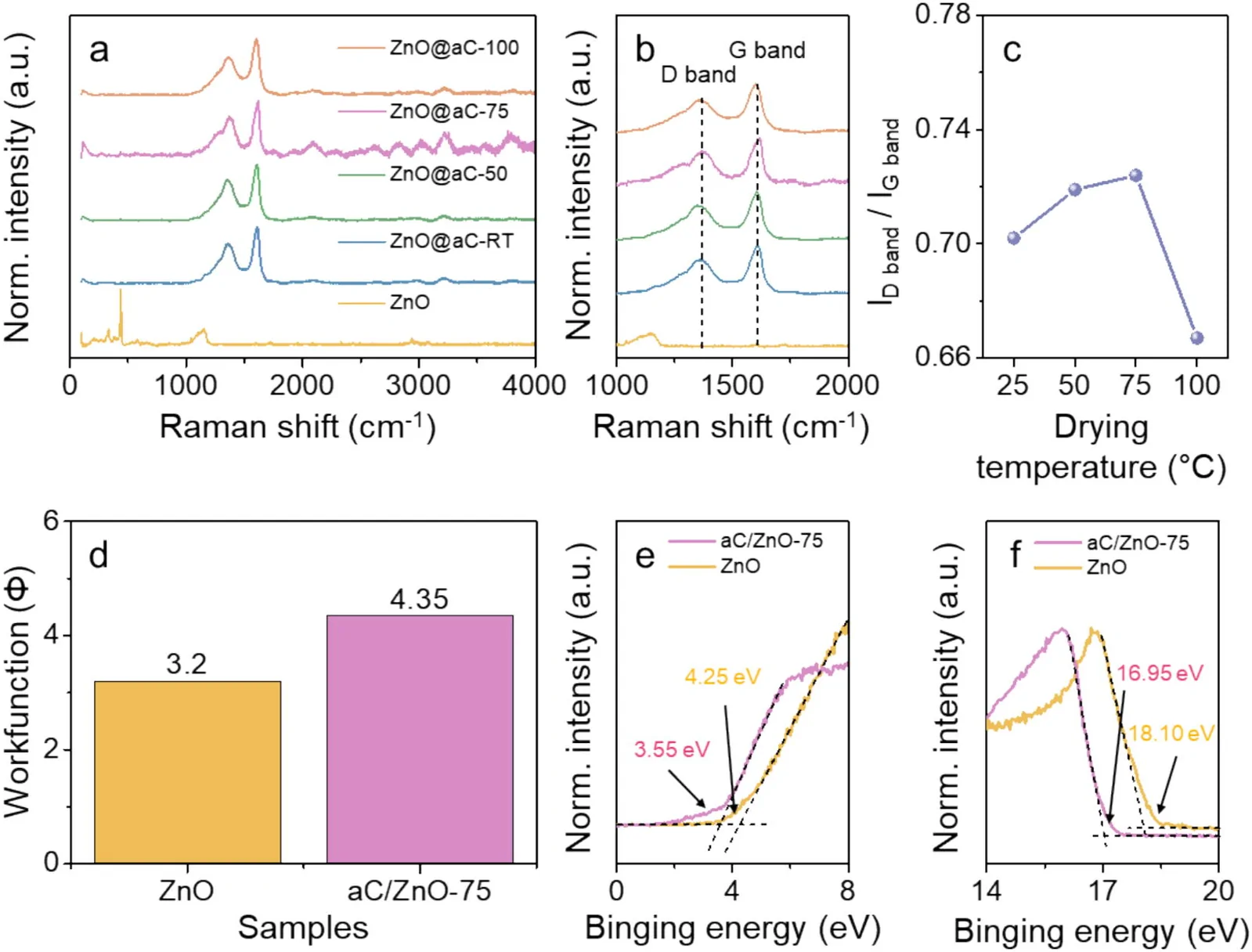
(a) Raman spectra of bare ZnO and aC/ZnO under different heat treatments; (b) Raman spectra in the 1000–2000 cm^−1^ range; (c) I_D_/I_G_ ratio versus heat treatment temperature; (d–f) comparison of (d) work function, (e) valence band maximum, (f) energy_cut-off_ for bare ZnO and aC/ZnO-75.

The energy distributions of the aC/ZnO PNSs were further analyzed through quantitative evaluation of the work function, Fermi level, and valence band maximum obtained by UPS. Fig. 7d and 7e compare the work function and valence band maximum of bare ZnO PNSs and aC/ZnO PNSs. Bare ZnO exhibits a work function of 3.2 eV. This value was calculated as 21.2 eV_(incident energy)_ – 18.10_(energy cut-off)_ + 0.1 eV_(correction value)_, as shown in Fig. 7d and 7f. The valence band maximum of bare ZnO is 4.25 eV. In contrast, aC/ZnO exhibits a higher work function of 4.35 eV. This value was calculated as 21.2 eV_(incident energy)_ – 16.95 eV_(energy cut-off)_ + 0.1 eV_(correction value)_, as shown in Fig. 7d and 7f. The valence band maximum of aC/ZnO is 3.55 eV, which is lower than that of bare ZnO, as shown in Fig. 7e. This shift indicates a transition from n-type behavior in ZnO PNSs to a more p-type characteristic in aC/ZnO, driven by interaction with aC. From a gas-sensing perspective, this change reduces the number of mobile carriers at the surface of the ZnO PNSs and leads to an increase in resistance.

To further assess the effect of aC, changes near the gas/aC surface and at the aC/ZnO interface were examined in comparison to bare ZnO, as illustrated in Fig. 8. The work function of the bare ZnO surface increases, which causes a downward shift of the Fermi level by 1.15 eV. In addition, the valence band maximum shifts downward by 0.45 eV at the interface. These changes lead to the formation of two distinct two-dimensional junctions, which ultimately enhance the gas-sensing response.

**Fig. 8:**
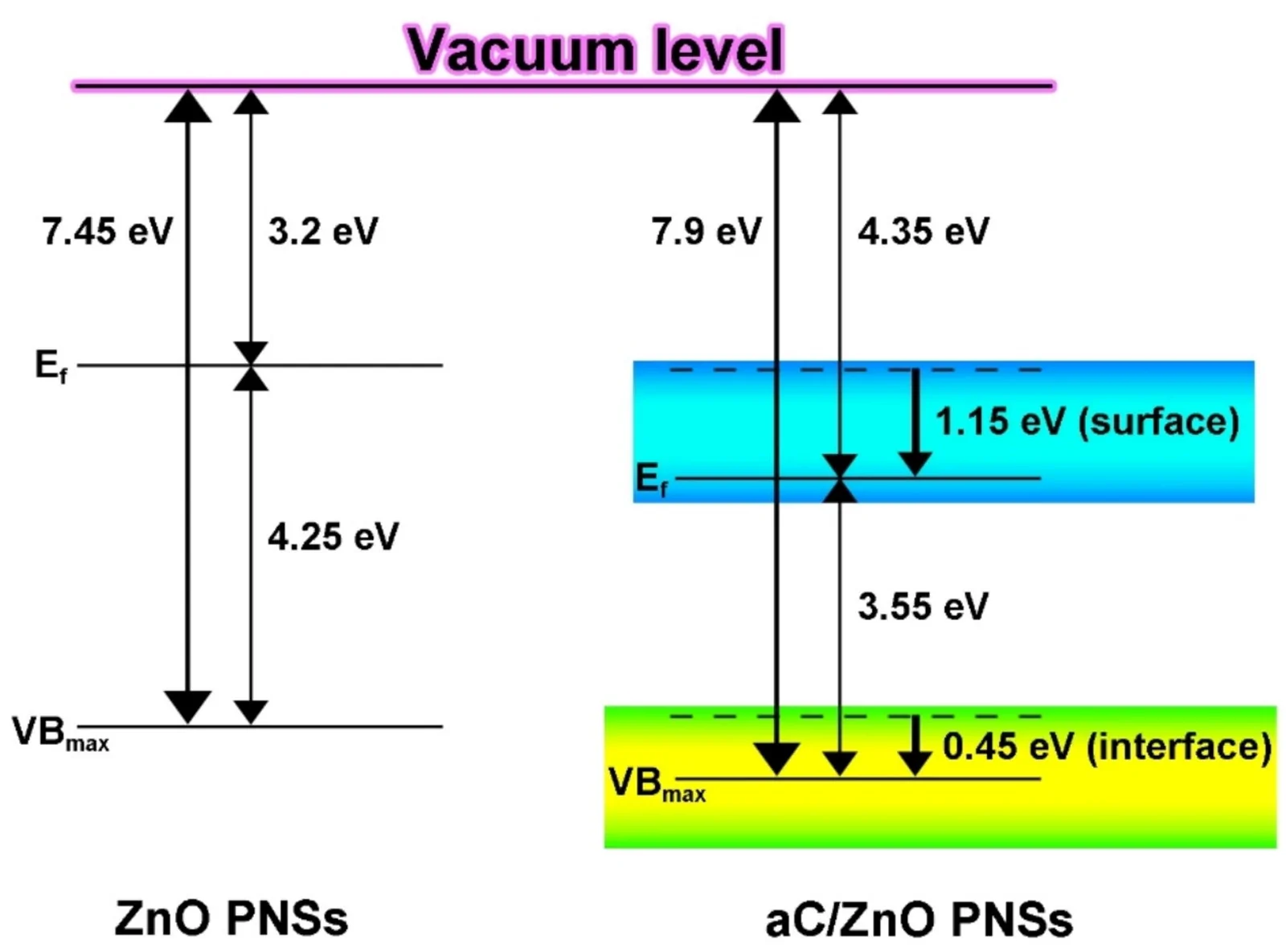
Changes in work function and valence band maximum at the surface and interface in aC/ZnO PNSs due to the introduction of aC.

### Various gas sensing features in aC/ZnO PNS

These energy distributions are highly relevant to gas-sensing behavior and enable the detection of NO_2_ at the ppb level. In the absence of target gas, both bare ZnO and aC/ZnO samples interact with oxygen ion species. The dominant oxygen species depend on the ambient temperature, as described by the following reactions [47], [48]:

(1) $${\text{O}}_{\text{2}(\text{gas})}↔{\;\text{O}}_{\text{2}(\text{ads})}$$

(2) $${\text{O}}_{\text{2}(\text{ads})}+{\;\text{e}}^{-}\to {{{\;\text{O}}_{\text{2}}}^{-}}_{\left(\text{ads}\right)}\left(\text{over 1}00^{{}^{\circ}}\text{C}\right)$$

(3) $${{{\text{O}}_{\text{2}}}^{-}}_{\left(\text{ads}\right)}+{\;\text{2e}}^{-}\to {{\;\text{2O}}^{-}}_{\left(\text{ads}\right)}\left(\text{between 1}00\;\text{and 3}00^{{}^{\circ}}\text{C}\right)$$

(4) $${O^{-}}_{\left(ads\right)}+e^{-}\to {O^{2-}}_{\left(ads\right)}\left(below300^{{}^{\circ}}C\right)$$

Oxygen has a high electron affinity [49] and induces the formation of an EDL on both bare ZnO and aC/ZnO samples by extracting surface electrons. As an n-type semiconductor, ZnO exhibits an internal conductive channel width (CCW) that is inversely related to the thickness of the EDL. When the EDL increases, the CCW decreases, resulting in higher resistance. Conversely, a thinner depletion layer leads to lower resistance, highlighting the interdependent relationship between these two parameters [50]. When NO_2_, a representative oxidizing gas, is introduced, it either extracts electrons from adsorbed oxygen ions or directly removes electrons from the ZnO surface in regions where oxygen ions are absent. This interaction further thickens the EDL. As a result, the CCW decreases and the resistance increases. As shown in Fig. 9, both bare ZnO and aC/ZnO samples exhibit measurable resistance changes at 200 °C, even at low NO_2_ concentrations ranging from 4 ppm to 200 ppb (Fig. 9a–9f, SI, Fig. S2). The initial resistance varies depending on the crystallinity of aC, which is influenced by post-heat treatment. Even under identical heat treatment conditions, the initial resistance may differ with changes in target gas concentration. This variation arises because, although the aC deposition method is identical, the coating thickness cannot be guaranteed to be exactly the same in every case. Therefore, the initial resistance should be considered within a certain range, as shown in Fig. 9a–9e. For comparison, NO_2_ gas-sensing results for aC without ZnO are presented in SI, Fig. S3. In this case, the response remains below 1.3 across all concentrations from 200 ppb to 4 ppm. These results indicate that bare aC or bare ZnO, each possessing only a surface, does not achieve the sensing performance of aC/ZnO, which features both a surface and an interface. The response time and recovery time range from 333.9 to 432.2 s and from 51 to 425 s, respectively, as shown in Fig. 9g and 9h. This behavior can be attributed to an increased number of electrons transitioning from the Fermi level to the conduction band as the operating temperature increases. In some cases, electron transitions from the valence band to the conduction band also increase. However, at low temperatures below 200 °C, sensing characteristics do not appear due to a lack of activated mobile carriers (SI, Fig. S4).

**Fig. 9:**
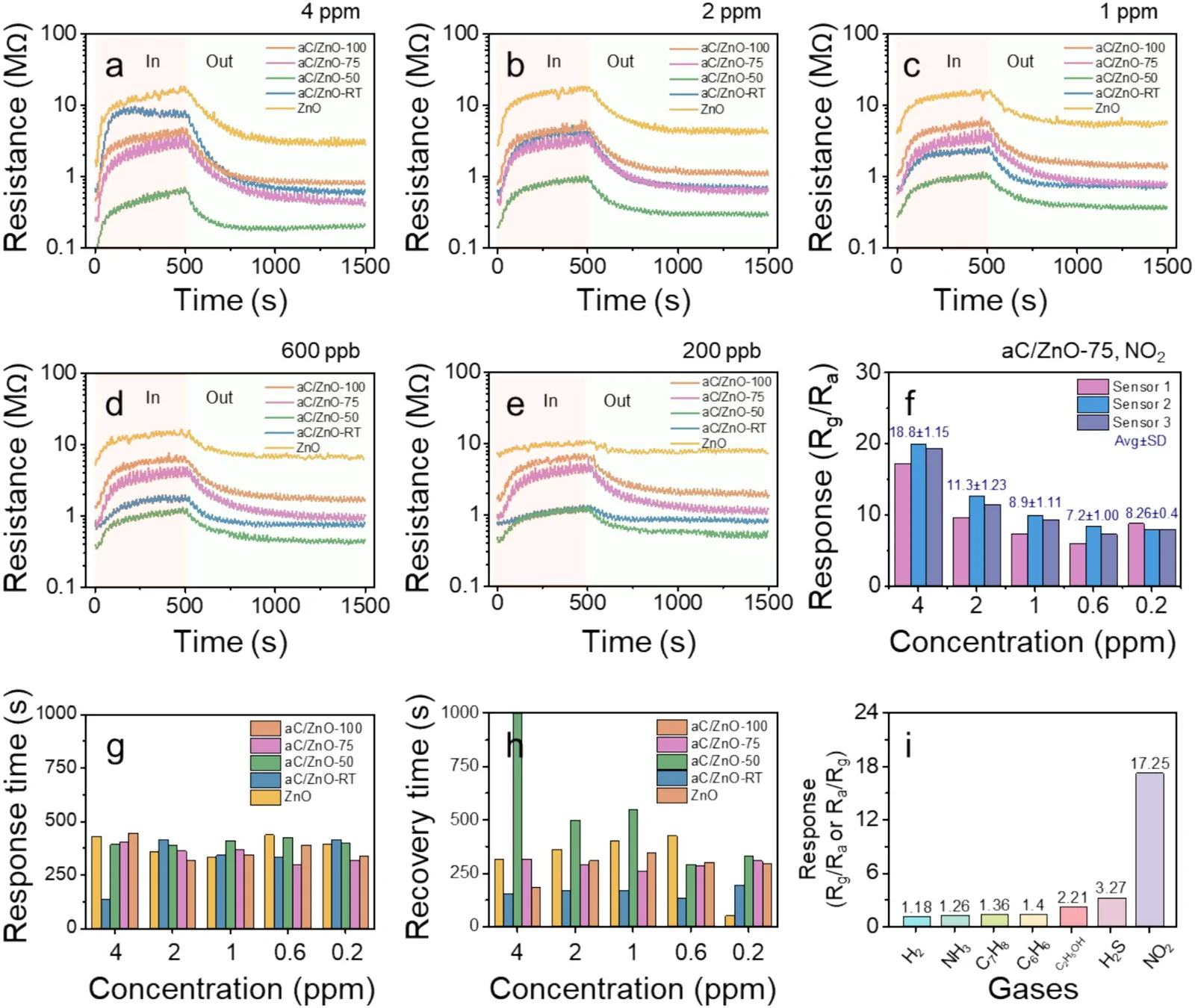
(a–e) NO_2_ resistance at 200 °C for different concentrations, (f) response, (g) response time, (h) recovery time; (i) selectivity for various gases in aC/ZnO-75.

For aC/ZnO PNSs heat treated at 75 °C using OSaCD on bare ZnO, the response at 200 °C ranges from 7.2 ± 1.0 to 18.8 ± 1.2. Regardless of sample type, increasing the operating temperature under all conditions leads to a significant change in the number of mobile carriers before and after target gas adsorption. This change results in an enhanced sensor response [51], [52]. This trend is particularly pronounced for oxidizing gases compared to high-concentration reducing gases, such as H_2_, NH_3_, C_7_H_8_, C_6_H_6_, C_2_H_5_OH, and H_2_S, as shown in Fig. 9i and SI, Fig. S5). In addition, higher relative humidity generally leads to reduced sensor responses. This reduction occurs because adsorption sites initially interact with water molecules, as shown in SI, Fig. S6) [53], [54]. In other words, as humidity increases, OH species become dominant on the surface. These species attract electrons less effectively than oxygen adsorbing species, which reduces the resistance change induced by NO_2_ gas. Ideally, a sensor response should involve a large resistance change before and after target gas adsorption. However, OH species create a screening effect that suppresses carrier movement. This effect gradually diminishes as the NO_2_ concentration decreases from 4 ppm to 200 ppb. To evaluate sensing reproducibility, eight repeated measurements were conducted at 200 °C for the lowest NO_2_ concentration of 200 ppb. A consistent response of 8.82 ± 0.23 was obtained, as shown in SI, Fig. S7(a). Furthermore, stable sensing performance was confirmed during a three-month long-term test at 200 °C for NO_2_ concentrations ranging from 4 ppm to 200 ppb, as shown in SI, Fig. S7(b).

### Reaction mechanisms at the surface and interface of aC/ZnO PNS

In contrast to bare ZnO, the introduction of aC enables simultaneous surface and interface engineering. Surface modification occurs at the aC/oxygen or aC/target gas interface, while interface modification occurs at the aC/ZnO junction. The presence of aC increases the number of gas adsorption sites due to a higher density of surface defects. First, at the oxygen/aC surface, as illustrated in Fig. 10, enhanced oxygen adsorption leads to increased electron withdrawal by oxygen species. This process results in a deeper EDL compared to that of bare ZnO. The deepened EDL increases the resistance and ultimately improves the sensor response. Second, at the aC/ZnO interface, a strong chemical bond forms between aC and ZnO. In bare ZnO, oxygen vacancies on the surface generate a large number of mobile carriers, which contribute to its n-type semiconductor behavior. These oxygen vacancies are associated with Zn^2+^ ions that are not bonded to oxygen. During the OSaCD process, the specimen is immersed in water, leading to the following reactions [55]:

(5) $${\text{Zn}}^{\text{2}+}+{\;\text{2OH}}^{-}\to \;\text{Zn}{\left(\text{OH}\right)}_{\text{2}}$$

(6) $$\text{Zn}{\left(\text{OH}\right)}_{\text{2}}\to \;\text{ZnO}\;+{\;\text{H}}_{\text{2}}\text{O}$$

**Fig. 10:**
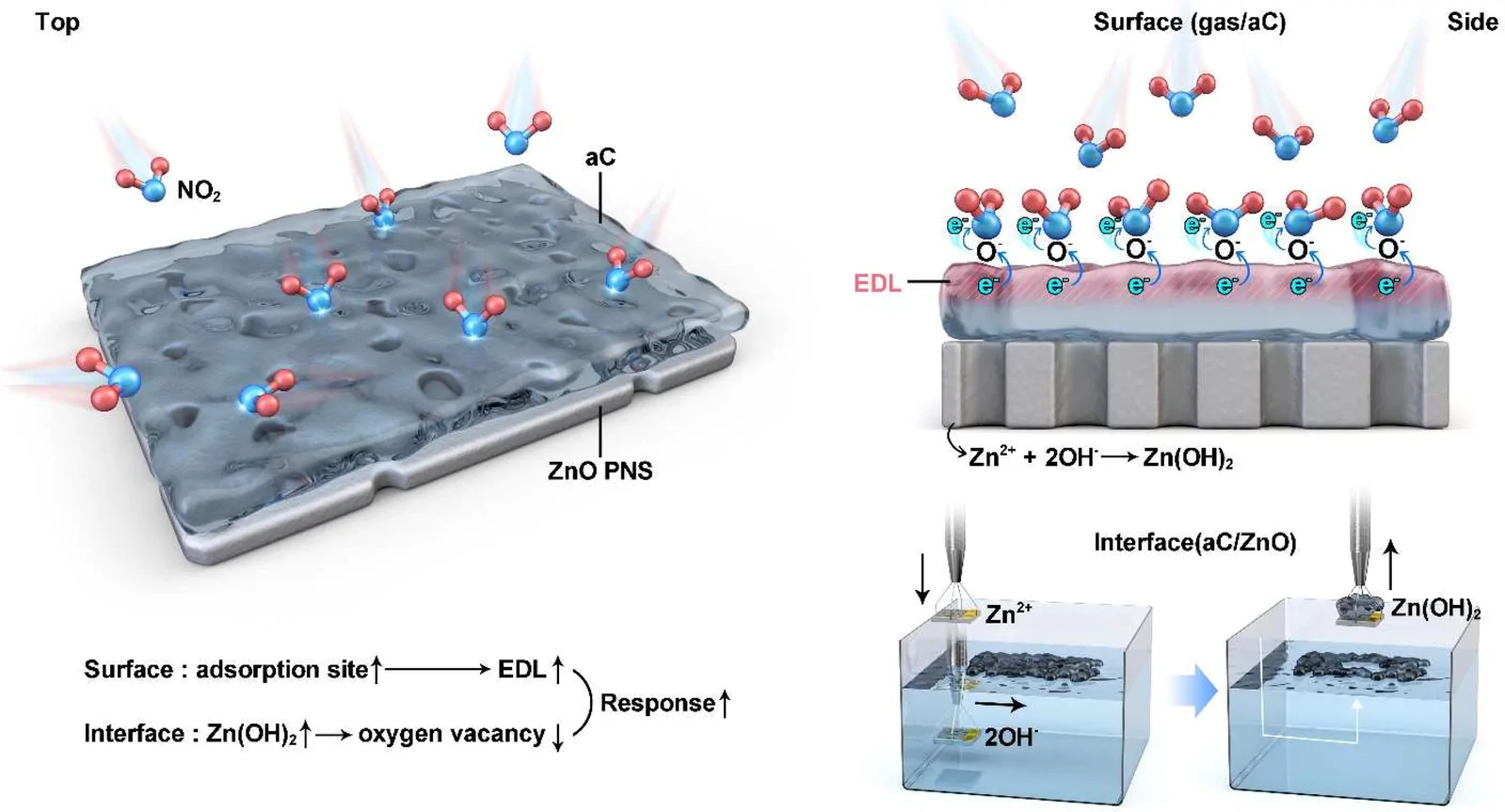
Conceptual diagram illustrating increased resistance at the surface and interface in aC/ZnO-75.

As shown in Fig. 10, Zn^2+^ ions react with OH^−^ ions to form an intermediate Zn(OH)_2_ phase. This intermediate subsequently transforms into ZnO and H_2_O during heat treatment. Before forming an interface with aC, the number of electrons associated with oxygen vacancies in bare ZnO decreases rapidly due to the formation of Zn(OH)_2_. This reduction lowers the concentration of mobile carriers and increases the resistance at the aC/ZnO interface. Therefore, the introduction of aC increases resistance by deepening the EDL at the surface and reducing oxygen vacancies at the interface. These effects together enhance the sensor response when resistance is measured.

This relationship can be more clearly understood by considering the Gibbs free energy change during the reaction process [56], [57]. As shown in Fig. 11, the introduction of aC into bare ZnO, indicated in black, generates a 1.15 eV junction near the surface, as shown in Fig. 8. This junction shifts the Gibbs free energy of the aC/ZnO PNSs reactant downward, from level ⑥ to a value between levels ④ and ⑤. Near the interface, a 0.45 eV junction is formed, as shown in Fig. 8. This junction shifts the activation energy of the aC/ZnO PNSs reactant upward, from the range of levels ⑧–⑨ to values greater than level ⑩. The synergistic effect of these two junctions enhances the sensor response, as reflected by the resistance change before and after gas adsorption.

**Fig. 11:**
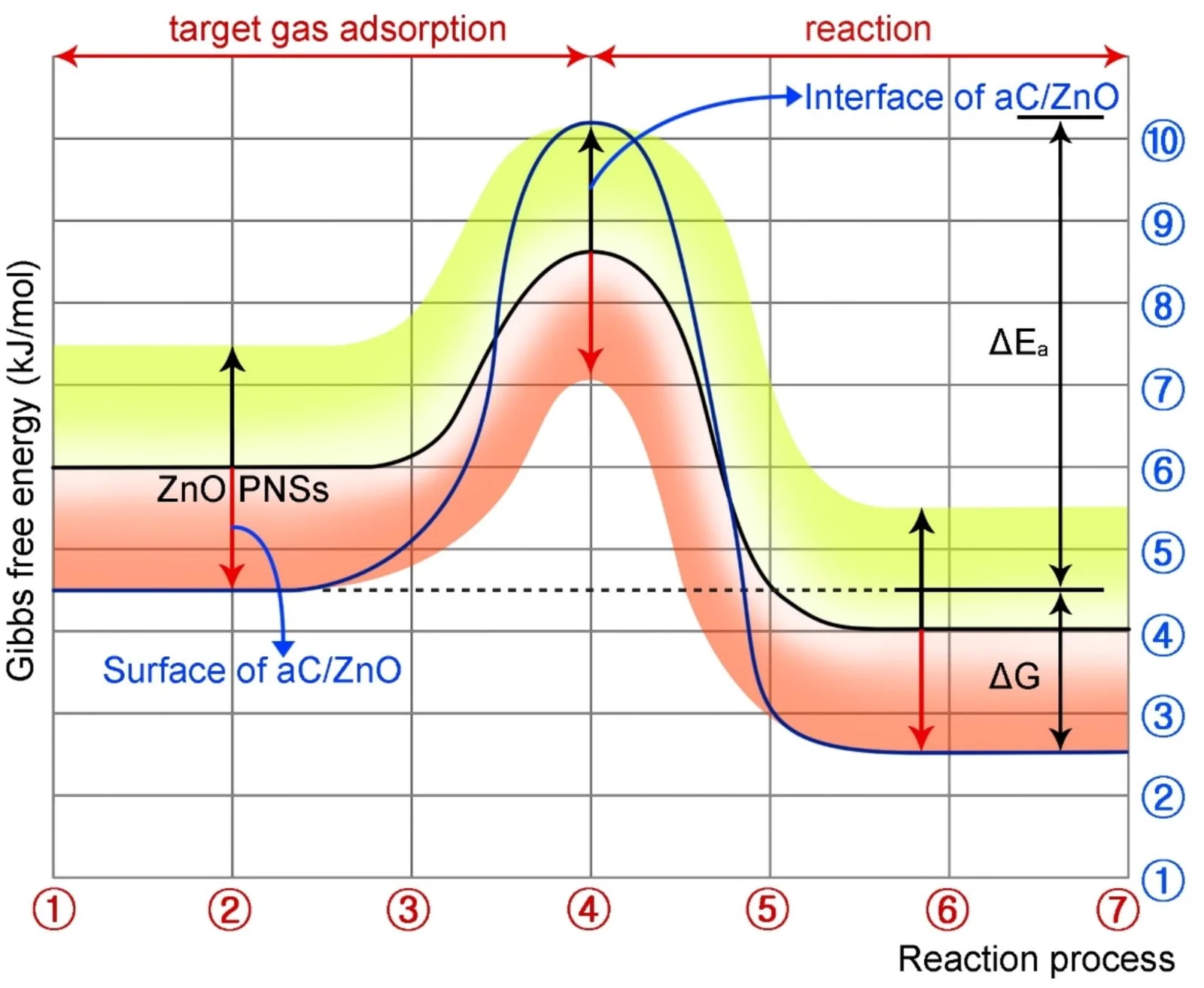
Schematic of Gibbs free energy differences at the surface and interface due to the introduction of aC in ZnO.

This gas-sensing mechanism differs significantly from commonly reported approaches, such as those based on nanograins or terrace, ledge, and kink (TLK) structures, which increase resistance by modifying cross-sectional areas [58], [59]. It also differs from mechanisms relying on heterojunctions, such as p–n or homojunctions including n–n or p–p junctions [60], [61], [62]. As discussed earlier, this mechanism is the first to suppress the role of oxygen vacancies at the n-type semiconductor interface while simultaneously expanding the EDL during the reaction of metal oxide semiconductors with surface oxygen. As a result, it enables synergistic effects through concurrent surface and interface engineering, exceeding the functionality of simple nanocomposites. In addition, the OSaCD technique employed in this study provides an effective surface modification method that can be applied to heterogeneous substrates by exploiting the fluidity of a liquid.

## Conclusions

In aC/ZnO PNSs prepared using the OSaCD technique, the aC layer with a thickness of 44.92 ± 28.46 nm, deposited by the FCVD method and heat treated at 75 °C, notably increased the depth of the surface EDL by promoting enhanced oxygen adsorption. At the same time, it reduced oxygen vacancies at the ZnO interface. This dual effect significantly increased the NO_2_ response to 18.8 ± 1.2 at 4 ppm and 200 °C. The improvement arises from the synergistic enhancement of gas-sensing performance at both the surface and the interface. A key advantage of the OSaCD technique is that it is not affected by the morphology, composition, or crystallinity of the sample. Therefore, based on the present study, which was limited to a fixed aC thickness, future research will focus on extending this approach to other oxide materials and identifying optimal aC thicknesses.

## Declaration of competing interest

The authors declare that they have no known competing financial interests or personal relationships that could have appeared to influence the work reported in this paper.

## References

[1] Baek J.W., Han S., Lee S.E., Ahn J., Park C., Nam J.S. (2024). Cobalt-Doped Ceria Sensitizer Effects on Metal Oxide Nanofibers: heightened Surface Reactivity for High-performing Chemiresistive Sensors. ACS Nano.

[2] Tang W., Chen Z., Song Z., Wang C., Wan Z., a., Chan, C. L. J., Chen, Z., Ye, W., Fan, Z. (2022). Microheater integrated nanotube array gas sensor for parts-per-trillion level gas detection and single sensor-based gas discrimination. ACS Nano.

[3] Martinelli E., Polese D., Catini A., D’Amico A., Di Natale C. (2012). Self-adapted temperature modulation in metal-oxide semiconductor gas sensors. Sens Actuators B.

[4] Xiong Y., Lin Y., Wang X., Zhao Y., Tian J. (2022). Defect engineering on SnO_2_ nanomaterials for enhanced gas sensing performances. Adv Powder Mater.

[5] Li C., Zhang D., Lei B., Han S., Liu X., Zhou C. (2003). Surface Treatment and Doping Dependence of In_2_O_3_ Nanowires as Ammonia Sensors. J Phys Chem B.

[6] Gu C., Shanshan L., Huang J., Shi C., Liu J. (2013). Preferential growth of long ZnO nanowires and its application in gas sensor. Sens Actuators B.

[7] Kim J.-H., Mirzaei A., Woo Kim H., Wu P., Kim S.S. (2019). Design of supersensitive and selective ZnO-nanofiber-based sensors for H_2_ gas sensing by electron-beam irradiation. Sens Actuators B.

[8] Nemufulwi M.I., Swart H.C., Mhlongo G.H. (2023). Highly selective acetone detection displayed by a surface engineered fiber-like ZnFe_2_O_4_ based-sensor following heat-treatment ramping rate variation. Mater Lett.

[9] Choi M.S., Na H.G., Bang J.H., Oum W., Choi S.-W., Kim S.S. (2019). Fast Semiconductor–Metal Bidirectional transition by Flame Chemical Vapor Deposition. ACS Omega.

[10] Jin C., Yang H., Lim T., Lee K.H., Lee W. (2024). NO_2_ gas-sensing enhancement by selective laser surface treatment of SnO_2_ nanorods. Sens Actuators B.

[11] Choi M.S., Mirzaei A., Na H.G., Kim S., Kim D.E., Lee K.H. (2021). Facile and fast decoration of SnO_2_ nanowires with Pd embedded SnO_2-x_ nanoparticles for selective NO_2_ gas sensing. Sens Actuators B.

[12] Ruey-Chi W., Chia-Chi H., Shu-Jen C. (2011). The evolution of well-aligned amorphous carbon nanotubes and porous ZnO/C core–shell nanorod arrays for photosensor applications. Nanotechnology.

[13] Zhao W., Li Y., Wang S., He X., Shang Y., Peng Q. (2014). Elastic improvement of carbon nanotube sponges by depositing amorphous carbon coating. Carbon.

[14] Teng Y., Mo M., Li Y., Xue J., Zhao H. (2018). Amorphous carbon-coated ZnO porous nanosheets: Facile fabrication and application in lithium- and sodium-ion batteries. J Alloy Compd.

[15] Kundu D., Banerjee D., Ghosh S., Das N.S., Thakur S., Das B. (2019). Plasma enhanced Chemical Vapour deposited amorphous carbon coating for hydrophobicity enhancement in commercial cotton fabrics. Physica E.

[16] Mori Y., Ueda M., Hashimoto M., Aoi Y., Tanase S., Sakai T. (2008). Amorphous carbon coated stainless separator for PEFCs. Surf Coat Technol.

[17] Zhang M., Li Z., Zhao J., Gong L., Meng A., Liu X. (2015). Amorphous carbon coating for improving the field emission performance of SiC nanowire cores. J Mater Chem C.

[18] Liu Y., Wiek A., Dzhagan V., Holze R. (2016). Improved electrochemical behavior of amorphous carbon-coated copper/CNT composites as negative electrode material and their energy storage mechanism. J Electrochem Soc.

[19] Show Y. (2007). Electrically conductive amorphous carbon coating on metal bipolar plates for PEFC. Surf Coat Technol.

[20] Kumari K., Kadian P., Kumar M., Randhawa J.K. (2024). Understanding Norfloxacin’s Turn-on Detection Mechanism: focusing on Amorphous Carbon-Coated Iron Oxide Nanoparticles. ACS Appl Opt Mater.

[21] Noh M., Kwon Y., Lee H., Cho J., Kim Y., Kim M.G. (2005). Amorphous Carbon-Coated Tin Anode Material for Lithium Secondary Battery. Chem Mater.

[22] Li Q., Li W., Feng Q., Wang P., Mao M., Liu J. (2014). Thickness-dependent fracture of amorphous carbon coating on SnO2 nanowire electrodes. Carbon.

[23] Wang Z., Tian W., Liu X., Yang R., Li X. (2007). Synthesis and electrochemical performances of amorphous carbon-coated Sn–Sb particles as anode material for lithium-ion batteries. J Solid State Chem.

[24] Guan W., Wang Y., Fischer C.B., Wehner S., Wang Z., Li J. (2020). Novel strategy to improve the tribological property of polymer: In-situ growing amorphous carbon coating on the surface. Appl Surf Sci.

[25] Zhang Y.J., Liu X.Y., Bai W.Q., Tang H., Shi S.J., Wang X.L. (2014). Magnetron sputtering amorphous carbon coatings on metallic lithium: Towards promising anodes for lithium secondary batteries. J Power Sources.

[26] Li K., Xu G., Wen X., Zhou J., Gong F. (2021). High-temperature friction behavior of amorphous carbon coating in glass molding process. Friction.

[27] Wang Y., Guan W., Fischer C.B., Wehner S., Dang R., Li J. (2021). Microstructures, mechanical properties and tribological behaviors of amorphous carbon coatings in-situ grown on polycarbonate surfaces. Appl Surf Sci.

[28] Xia T., Zhang W., Wang Z., Zhang Y., Song X., Murowchick J. (2014). Amorphous carbon-coated TiO_2_ nanocrystals for improved lithium-ion battery and photocatalytic performance. Nano Energy.

[29] Kim J.S., Halim M., Byun D., Lee J.K. (2014). Amorphous carbon-coated prickle-like silicon of micro and nano hybrid anode materials for lithium-ion batteries. Solid State Ion.

[30] Ge Y., Jiang H., Zhu J., Lu Y., Chen C., Hu Y. (2015). High cyclability of carbon-coated TiO_2_ nanoparticles as anode for sodium-ion batteries. Electrochim Acta.

[31] Shin D.H., Lee J.S., Jun J., Jang J. (2014). Fabrication of amorphous carbon-coated NiO nanofibers for electrochemical capacitor applications. J Mater Chem A.

[32] Wang H., Dai M., Li Y., Bai J., Liu Y., Li Y. (2021). The influence of different ZnO nanostructures on NO_2_ sensing performance. Sens Actuators B.

[33] Li S., Yu L., Cao L., Zhang C., Du H., Wang H. (2024). Engineering of Thickness Tunable 2D Graphdiyne Film to ZnO Nanowalls via Nanospace‐Confined Synthesis Promotes NO_2_ Gas Sensing Performance. Sens Actuators B.

[34] Gao R., Gao L., Zhang X., Gao S., Xu Y., Cheng X. (2021). The controllable assembly of the heterojunction interface of the ZnO@rGO for enhancing the sensing performance of NO_2_ at room temperature and sensing mechanism. Sens Actuators B.

[35] Yuan M., Peng C., Fu J., Liu X., Wang Z., Xu S. (2022). High surface area ZnO/rGO aerogel for sensitive and selective NO_2_ detection at room temperature. J Alloy Compd.

[36] Li Q., Cui Y., Lin J., Zhao C., Ding L. (2022). Synthesis of carbon microsphere-assisted snowflake-like ZnO nanomaterials for selective detection of NO_2_ at room temperature. J Ind Eng Chem.

[37] Kwon Y.J., Mirzaei A., Kang S.Y., Choi M.S., Bang J.H., Kim S.S. (2017). Synthesis, characterization and gas sensing properties of ZnO-decorated MWCNTs. Appl Surf Sci.

[38] Park S., Byoun Y., Kang H., Song Y.-J., Choi S.-W. (2019). ZnO nanocluster-functionalized single-walled carbon nanotubes synthesized by microwave irradiation for highly sensitive NO_2_ detection at room temperature. ACS Omega.

[39] Yin L., Liu C., Yu T., Feng J., Zhu G., Zeng H. (2024). Electrospun ZnO nanotubes decorated with zinc ferrite nanoparticles as sensing material for hydrogen sulfide detection. ACS Appl Nano Mater.

[40] Cheng X.-L., Xu Q.-Q., Li S.-S., Li J., Zhou Y., Zhang Y. (2021). Oxygen vacancy enhanced Co_3_O_4_/ZnO nanocomposite with small sized and loose structure for sensitive electroanalysis of Hg(II) in subsidence area water. Sens Actuators B.

[41] Shi D., Ji Y., Lu F., Yao J., Pei L. (2023). Construction of a NiFe-LDH catalyst with a three-dimensional unified gas diffusion layer structure via a facile acid etching route for the oxygen evolution reaction. Mater Chem Front.

[42] Yang Y., Wu S., Cao Y., Li S., Xie T., Lin Y. (2022). A highly efficient room-temperature formaldehyde gas sensor based on a Ni-doped ZnO hierarchical porous structure decorated with NiS illuminated by UV light. J Alloy Compd.

[43] Yu J., Shang Q., Zhang M., Hu L., Zhou Y. (2024). Bio-based superhydrophobic coatings from cashew nut for self-cleaning and long-term anticorrosion applications. Prog Org Coat.

[44] Zhao S., Li C., Liu J., Liu N., Qiao S., Han Y. (2015). Carbon quantum dots/SnO_2_–Co_3_O_4_ composite for highly efficient electrochemical water oxidation. Carbon.

[45] Zhu Y., Tan Y., Li H. (2023). N, O co-doped carbon nanosheets synthesized based on polydopamine in situ grown on C_3_N_4_ template for supercapacitors. J Storage Mater.

[46] Fu H., Zhang J., Zhao L., Huang Y., Chen B. (2024). Investigations of NO reduction by coal-based activated carbon with KOH activation: Performance and mechanism. Chemosphere.

[47] Barsan N., Weimar U. (2001). Conduction Model of Metal Oxide Gas Sensors. J Electroceram.

[48] Cheng P., Lv L., Wang Y., Zhang B., Zhang Y., Zhang Y. (2021). SnO_2_/ZnSnO_3_ double-shelled hollow microspheres based high-performance acetone gas sensor. Sens Actuators B.

[49] Li J., Na E., Liang X., Liang Q., Fan M., Chen H. (2024). Surface oxygen chemistry of metal oxide semiconductors for gas-sensing applications. Inorg Chem Front.

[50] Lim J.-C., Jin C., Choi M.S., Kim M.Y., Kim S.-I., Choi S.-M. (2021). Synthesis, morphology, characterisation, and ethanol gas sensing of hierarchical flower-like Co-doped WO_3_ nanoplates by solvothermal route. Ceram Int.

[51] Kim D., Kim D.H., Cho H., Kim Y., Mun C.S., Kim G.-T. (2025). Gate-Modulated Temperature-Dependent NO_2_ Gas Responsivity of h-BN Bottom-Gated MoS_2_ Field-effect Transistor. ACS Appl Electron Mater.

[52] Li J., Wang N., Yang X., Li J., Xiong S., Fu H. (2025).

[53] Li Y., Song Z., Li Y., Chen S., Li S., Li Y. (2019). Hierarchical hollow MoS_2_ microspheres as materials for conductometric NO_2_ gas sensors. Sens Actuators B.

[54] Qiu J., Hu X., Shi L., Fan J., Min X., Zhang W. (2021). Enabling selective, room-temperature gas detection using atomically dispersed Zn. Sens Actuators B.

[55] Mosayebi E., Azizian S., Hajian A. (2015). Synthesis of nanostructured and microstructured ZnO and Zn(OH)_2_ on activated carbon cloth by hydrothermal and microwave-assisted chemical bath deposition methods. Superlattice Microst.

[56] Bartel C.J., Millican S.L., Deml A.M., Rumptz J.R., Tumas W., Weimer A.W. (2018). Physical descriptor for the Gibbs energy of inorganic crystalline solids and temperature-dependent materials chemistry. Nat Commun.

[57] Sangeetha A., Shanmugan S., Gorjian S. (2022). Experimental evaluation and thermodynamic Gibbs free energy analysis of a double-slope U-shaped stepped basin solar still using activated carbon with ZnO nanoparticles. J Clean Prod.

[58] Han X., Jin M., Xie S., Kuang Q., Jiang Z., Jiang Y. (2009). Synthesis of Tin Dioxide Octahedral Nanoparticles with Exposed High-Energy 221 Facets and Enhanced Gas-Sensing Properties. Angew Chem Int Ed.

[59] Taulo G.T., Shaalan N.M., Mohamed G.G., Ayad M.M., El-Moneim A.A. (2024). Inkjet printing of SnO_2_ nanoparticles with exposed high-energy facets for CO gas sensing. Ceram Int.

[60] Zhao S., Shen Y., Zhou P., Li G., Hao F., Han C. (2020). Construction of ZnO–SnO_2_ n-n junction for dual-sensing of nitrogen dioxide and ethanol. Vacuum.

[61] Liu X., Hu M., Wang Y., Liu J., Qin Y. (2016). High sensitivity NO_2_ sensor based on CuO/p-porous silicon heterojunction at room temperature. J Alloy Compd.

[62] Zheng W., Xu Y., Zheng L., Yang C., Pinna N., Liu X. (2020). MoS_2_ Van der Waals p–n Junctions Enabling Highly Selective Room-Temperature NO_2_ Sensor. Adv Funct Mater.

