# Supplementary material for: Simultaneous engineering of the surface (oxygen/amorphous carbon) and interface (amorphous carbon/ZnO) of ZnO using a one-spoon amorphous carbon deposition technique
Source: J Adv Res. 2026 Jan 30;88:731–42. doi: 10.1016/j.jare.2026.01.057 (PMC13669916; doi:10.1016/j.jare.2026.01.057)
Supplement: Supplementary file 1 [file mmc1.docx]

Supplementary Information

**Simultaneous engineering of the surface (oxygen/amorphous carbon) and interface (amorphous carbon/ZnO) of ZnO using a one-spoon amorphous carbon deposition technique**

**Table S1.** Types and area (%) of oxygen on the surface of each sample.

|  | ZnO | aC/ZnO-RT | aC/ZnO-50 | aC/ZnO-75 | aC/ZnO-100 |
| --- | --- | --- | --- | --- | --- |
| Oxygen lattice | 71.3 % | 59.4 % | 74.2 % | 73.3 % | 68.7 % |
| Oxygen vacancies | 22.7 % | 28.5 % | 17.8 % | 19.9 % | 25.1 % |
| Chemisorbed oxygen | 5.9 % | 12.1 % | 8.0 % | 6.8 % | 6.2 % |

**Figure S1.** Thickness of aC layer (44.92±28.46 nm) obtained from six different aC/ZnO PNSs.

**Figure S2.** Changes in sensor response at different NO_2_ gas concentrations measured at 200 °C.

**Figure S3.** (a) NO_2_ resistance and (b) corresponding response at 200 °C for various concentrations in bare aC-75.

**Figure S4.** Resistance changes of aC/ZnO-75 samples at different NO_2_ concentrations below 200 °C. (a) RT, (b) 50 °C, (c) 100 °C, (d) 150 °C.

**Figure S5.** Comparison of responses to different gases at 200 °C and a concentration of 10 ppm.

**Figure S6.** Sensor response variations with humidity for different NO_2_ concentrations: (a) 4 ppm, (b) 2 ppm, (c) 1 ppm, (d) 600 ppb, (e) 200 ppb, and (f) 200 ppb–4 ppm. Sensor operating temperature: 200 °C; ambient humidity: 22 °C. NO_2_ gas with 90%, 50%, and 30% RH at 22 °C was injected.

**Figure S7.** (a) Repeatability over eight measurements and (b) long-term stability over 3 months for 0.2 ppm NO_2_ at 200 °C in the aC/ZnO-75 sample.
